# Multiparametric magnetic resonance imaging for the differential diagnosis between granulomatous prostatitis and prostate cancer: a literature review to an intriguing diagnostic challenge

**DOI:** 10.3389/fonc.2023.1178430

**Published:** 2023-06-05

**Authors:** Caterina Gaudiano, Benedetta Renzetti, Cristina De Fino, Beniamino Corcioni, Federica Ciccarese, Lorenzo Bianchi, Riccardo Schiavina, Matteo Droghetti, Francesca Giunchi, Eugenio Brunocilla, Michelangelo Fiorentino

**Affiliations:** ^1^ Department of Radiology, IRCCS Azienda Ospedaliero-Universitaria di Bologna, Bologna, Italy; ^2^ Department of Medical and Surgical Sciences (DIMEC), University of Bologna, Bologna, Italy; ^3^ Division of Urology, IRCCS Azienda Ospedaliero-Universitaria di Bologna, Bologna, Italy; ^4^ Department of Pathology, IRCCS Azienda Ospedaliero-Universitaria di Bologna, Bologna, Italy; ^5^ Department of Medical and Surgical Sciences, University of Bologna, Bologna, Italy

**Keywords:** prostate cancer, granulomatous prostatitis, non-specific granulomatous prostatitis, granulomatous prostatitis induced by BCG, multiparametric magnetic resonance imaging, PI-RADS score

## Abstract

Multiparametric magnetic resonance imaging (mpMRI) is currently the most effective diagnostic tool for detecting prostate cancer (PCa) and evaluating adenocarcinoma-mimicking lesions of the prostate gland, among which granulomatous prostatitis (GP) represents the most interesting diagnostic challenge. GP consists of a heterogeneous group of chronic inflammatory lesions that can be differentiated into four types: idiopathic, infective, iatrogenic, and associated with systemic granulomatous disease. The incidence of GP is growing due to the increase in endourological surgical interventions and the adoption of intravesical instillation of Bacillus Calmette-Guerin in patients with non-muscle invasive bladder cancer; therefore, the difficulty lies in identifying specific features of GP on mpMRI to avoid the use of transrectal prostate biopsy as much as possible.

## 1 Introduction

There has recently been increasing interest in the diagnostic impact of multiparametric magnetic resonance imaging (mpMRI), which has become crucial for detecting prostate cancer (PCa) in both the peripheral and transition zones (PZ and TZ) and evaluating adenocarcinoma-mimicking lesions of the prostate gland, before planning a transrectal ultrasound (TRUS)/MRI fusion targeted biopsy (1).

As a matter of fact, a valid assessment of suspicious areas in mpMRI is a determinant for avoiding biopsy in patients without target lesions, for averting errors of overdiagnosis related to random biopsies, and for differentiating tumor-like lesions from PCa, which can be monitored in a periodic follow-up (2, 3).

Of the adenocarcinoma-mimicking lesions of the prostate gland, granulomatous prostatitis (GP) usually has the same clinical appearance as PCa, with obstructive and/or irritative symptoms, a diffuse or focal enlargement of the gland at digital rectal examination, and increasing levels of prostate-specific antigen (PSA) (4).

GP comprises a heterogeneous group of chronic inflammatory lesions of often unknown etiology and pathogenesis usually occurring in the PZ (5), which are relatively rare, accounting for 3.3% of all benign conditions of the gland (6), and are the best tumor mimickers.

Nevertheless, the incidence of GP is growing due to the increase of endourological surgical interventions and the adoption of intravesical instillation of Bacillus Calmette-Guerin (BCG) in patients with non-muscle invasive bladder cancer (NMIBC) (6, 7).

The GP is classified into four types, based on etiopathogenetic entities (**Table 1**) and the corresponding histopathological findings (**Table 2**), which we will analyze in detail.

**Table 1:** Etiological classification of granulomatous prostatitis.

**Idiopathic**	• Typical non-specific granulomatous prostatitis • Xanthogranulomatous prostatitis
**Infective**	• After Bacillus Calmette-Guerin vesical instillations • Bacterial (Tuberculosis, Brucellosis, Syphilis) • Fungal (Coccididiomycosis, Cryptococcosis, Blastomycosis, Histoplasmosis, Paracoccidioidomycosis) • Parasitic (Schistosomiasis, Echinococcosis, Enterobiasis) • Viral (Herpes simplex virus)
**Iatrogenic**	• Post-surgical (TURP) • Actinic
**Associated with other rare systemic granulomatous diseases**	• Sarcoidosis, rheumatoid arthritis • Wegener’s granulomatosis • Polyarteritis nodosa, Churg–Strauss syndrome

TURP, transurethral resection of the prostate.

**Table 2:** Histopathological features of granulomatous prostatitis types.

GP types	Histopathological features
**Idiopathic**	• Non-caseous granulomas with a periglandular distribution, consisting of epithelioid cells, neutrophils, histiocytes, lymphocytes, and desquamated cells. Dilated ducts are usually observed • In the xanthogranulomatous subtype, focal accumulations of cholesterol-laden histiocytes are usually seen in the prostate
**Infective**	• Confluent foci of well-formed caseous granulomas with Langhan’s-type giant cell, surrounded by epithelioid histiocytes • In BCG-related GP, caseating or noncaseating granulomas are also seen, although acid-fast bacilli are variably present; Ziehl–Neelsen stain can be useful in obtaining a final diagnosis
**Iatrogenic**	• Dense inflammation of the prostatic stroma, usually with the presence of rheumatoid-like nodules consisting of palisading histiocytes with foci of fibrinoid necrosis
**Associated with other rare systemic granulomatous diseases**	• Depending on the primary granulomatous disease

GP, granulomatous prostatitis; BCG, Bacillus Calmette-Guerin.

Regarding the mpMRI features of GP, large series studies are not available due to the rarity of the disease; therefore, in the literature, mainly case reports and small case series are reported. Nevertheless, in this paper, we intend to illustrate both the most common and the least frequent features of the various types of GP on mpMRI, through a narrative literature review, in order to highlight any radiological criteria for the differential diagnosis between this inflammatory condition and PCa and avoid as much as possible the use of TRUS prostate biopsy. Particularly, we want to focus on evaluating the GP features on the multiparametric study protocol, including T2-weighted (T2w), diffusion-weighted imaging (DWI) with apparent diffusion coefficient (ADC) map, and dynamic contrast-enhanced (DCE) sequences.

## 2 Materials and methods

This literature review was conducted by searching on PubMed the following keywords: “granulomatous prostatitis” AND “MRI” OR “mpMRI” OR “multiparametric magnetic resonance imaging” OR “magnetic resonance imaging” and including articles published from 2002 to 2022.

The bibliographic search produced 46 results.

**Figure 1:**
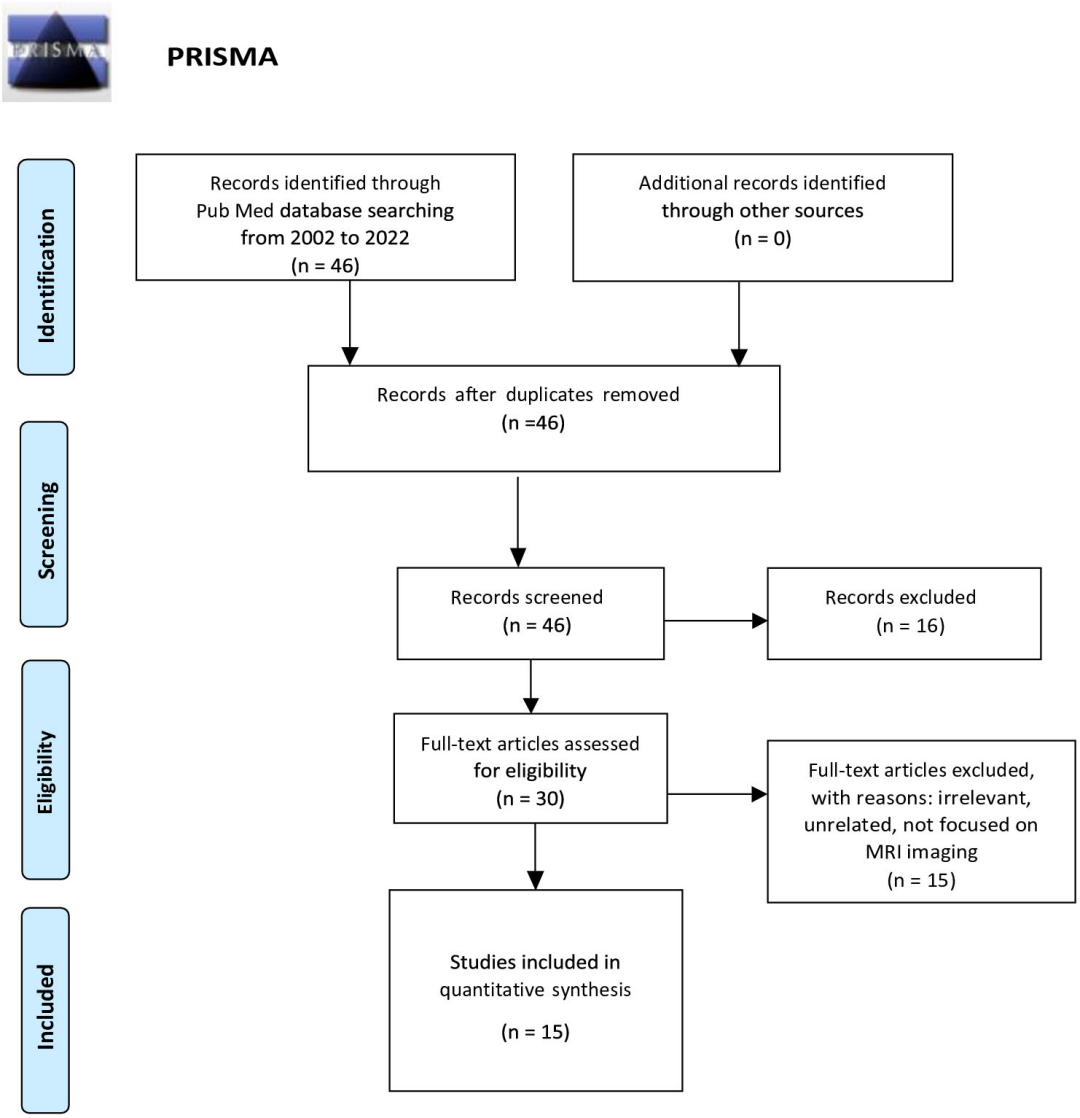
Preferred reporting items for systematic reviews and meta-analyses (PRISMA) flow diagram.

After a reading of the titles and abstracts, articles that did not focus on the characteristics of GP on mpMRI were eliminated, the 30 remaining articles were read thoroughly, and, after the elimination of repetitive, irrelevant, and unrelated articles, 15 publications remained (**Figure 1**).

The selected articles were the ones focused on the peculiar characteristics, and other main variants, of the different types of GP on mpMRI, in the perspective of the differential diagnosis with PCa.

We have attempted to include a variety of articles to ensure a comprehensive and exhaustive assessment of the various forms of GP, selecting the articles focused on mpMRI appearance with a preference for case series rather than case reports.

We prioritized papers with a protocol study adhering as much as possible to the Prostate Imaging Reporting and Data System (PI-RADS) guidelines, including high-resolution T2w in the axial, sagittal, and coronal planes; T1w in the axial plane; and multi-b values and high-b value DWI, with the corresponding ADC map and DCE sequences.

In an article (8), it was specified that, being a retrospective analysis, the imaging parameters were not standardized; thus, 5, 4, and 1 of the 10 patients underwent a spin-echo T1w, gradient-echo T1w, and unenhanced T1w, respectively, while the other sequences were the same. For the same reason, in another article (9), the images after infusion of gadolinium were not available.

**Table 3** shows the detailed description of the technical equipment used by each author group.

**Table 3:** Detailed description of the technical equipment used by each author group.

Authors	Scanner	Endorectal coil	Study protocol	PI-RADS score
Kitzing YX et al. (4)	3 T	No	T2, T1, multi b-DWI, ADC maps and DCE	≥3
Bertelli E et al. (5)	1.5 T	No	T2, T1, multi b-DWI, ADC maps and DCE	≥3
Crocetto F et al. (6)	Not reported	Not reported	Not reported	≥3
Suzuki T et al. (8)	1.5 T	No	T2, T1, multi b-DWI, ADC maps (no DCE)	Not reported
Wang Z et al. (9)	3 T	No	T2, T1, multi b-DWI, ADC maps (no DCE)	Not reported
Cheng Y et al. (10)	Not reported	Not reported	T2, T1, multi b-DWI, ADC maps, and DCE	Not reported
Lee SM et al. (11)	1.5 T	No	T2, T1, multi b-DWI, ADC maps, and DCE	≥3
Gottlieb et al. (12)	3 T	No	T2, T1, multi b-DWI, ADC maps, and DCE	≥3
Bour L et al. (13)	1.5 T	Yes (4/5 patients)	T2, T1, multi b-DWI, ADC maps, and DCE	Not reported
Rais-Bahrami S et al. (14)	3 T	No	T2, T1, multi b-DWI, ADC maps, and DCE	≥3
Kawada H et al. (15)	1.5 T	No	T2, T1, multi b-DWI, ADC maps, and DCE	Not reported
Han C et al. (16)	Not reported	No	T2, T1, multi b-DWI, ADC maps, and DCE	≥3
Suditu N et al. (17)	Recommended 3 T	Recommended	T2, T1, multi b-DWI, ADC maps, and DCE	Not reported
Lee S et al. (18)	3 T	No	T2, T1, multi b-DWI, ADC maps, and DCE	≥3 (except type C₌1)
De Luca L et al. (19)	3 T	No	T2, T1, multi b-DWI, ADC maps, and DCE	≥3

PI-RADS, Prostate Imaging Reporting and Data System; DWI, diffusion-weighted imaging; ADC, apparent diffusion coefficient; DCE, dynamic contrast enhanced.

In all the articles, it was emphasized that the confirmatory diagnosis was histopathological through a target or random sub head prostate biopsy, even if a central pathological review was not specified.

In five articles (8, 9, 12, 15, 18), it has been underlined that mpMRI imaging wax interpreted by two radiologists with many years of post-training experience (from a minimum of 3 years to a maximum of 18 years), and in another article (14), it has been emphasized that diagnostic mpMRI studies were subjected to radiological evaluation at a multidisciplinary conference on prostate imaging.

The findings obtained in the articles are summarized in **Table 4** and described in detail in the following sections.

**Table 4:** Multiparametric MRI features of the various types of granulomatous prostatitis.

Non-specific granulomatous prostatitis (NSGP)			
Sequences	Typical pattern	Less frequent patterns	Subtype: Xantogranulomatous prostatitis
**T2w**	Hypointense circumscribed nodule in the PZ Diameter < 1.5 cm	Hypointense nodule Diameter between 1 and 3.5 cm Both the PZ and TZ The involvement of only the TZ is an exception	Hypointensity with patchy or diffuse involvement of the PZ associated with disappearance of the normal demarcation between PZ and TZ
**DWI**	Hyperintensity	Hyperintensity	Hyperintensity
**ADC map**	Low ADC value	Low ADC value	Low ADC value
**DCE**	Moderate hyperenhancement	High and early enhancement followed by early wash-out	Diffuse hyperenhancement
Specific granulomatous prostatitis (infective and iatrogenic)			
Sequences	Diffuse pattern	Nodular pattern	Cystic pattern
**T2w**	Diffuse, heterogeneous, non-circumscribed, hypointensity of the PZ Frequent extension in the TZ Presence of capsular bulging	Solid nodule, polygonal in shape, markedly hypointense Diameter > 1.5 cm	Hyperintense nodule with peripheral hypointensity Central necrosis (caseation)
**DWI**	Hyperintensity	Hyperintensity	Hyperintensity
**ADC map**	Low ADC value	Low ADC value	Low ADC value
**DCE**	Moderate or marked enhancement	Contrast enhancement inhomogeneous, early, and prolonged	Early and prolonged peripheral rim enhancement with avascular core (“ring enhancement pattern”)
Associated with systemic granulomatous disease			
Sequences			
**T2w**	Hypointensity		
**DWI**	Hyperintensity		
**ADC map**	Low ADC value		
**DCE**	Diffuse contrast enhancement		

DWI, diffusion-weighted imaging; ADC, apparent diffusion coefficient; DCE, dynamic contrast enhanced; PZ, peripheral zone; TZ, transition zone.

### 2.1 Multiparametric MRI features and histopathological correlation

Based on histopathological findings and underlying etiology, the GP is classified into four types:

1. Idiopathic (non-specific and non-necrotic)
2. Infective (specific, non-necrotic or necrotic)
3. Iatrogenic (post-surgical)
4. Associated with systemic granulomatous disease (4)

#### 2.1.1 Idiopathic granulomatous prostatitis

Non-specific granulomatous prostatitis (NSGP) represents the most common type among GP, accounting for approximately 60%–77.7% (6).

The etiology of NSGP is unknown, but some studies have hypothesized an autoimmune reaction to cell debris, secretion spilling, and bacterial toxins into the stroma, resulting in blockage of the ducts and reflux of urine (10).

Histologically, NSGP is characterized by histiocytoid granulomas with clusters of macrophages, intermingled with multinucleated giant cell, lymphocytes, plasma cells, and neutrophils. The multinucleated giant cells might be absent (6) (**Table 2**).

**Figure 2:**
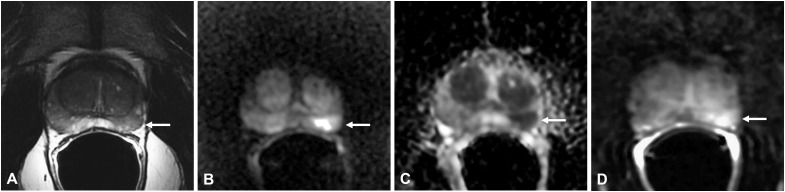
Non-specific granulomatous prostatitis in a 56-year-old patient with a PSA value of 6 ng/ml. The axial T2-weighted sequence shows a hypointense nodule in the left mid-peripheral zone (PZ) (arrow in **A**) with high hyperintensity on the DWI image (arrow in **B**), marked hypointensity on the ADC map (arrow in **C**), and high contrast enhancement on the DCE image (arrow in **D**).

**Figure 3:**
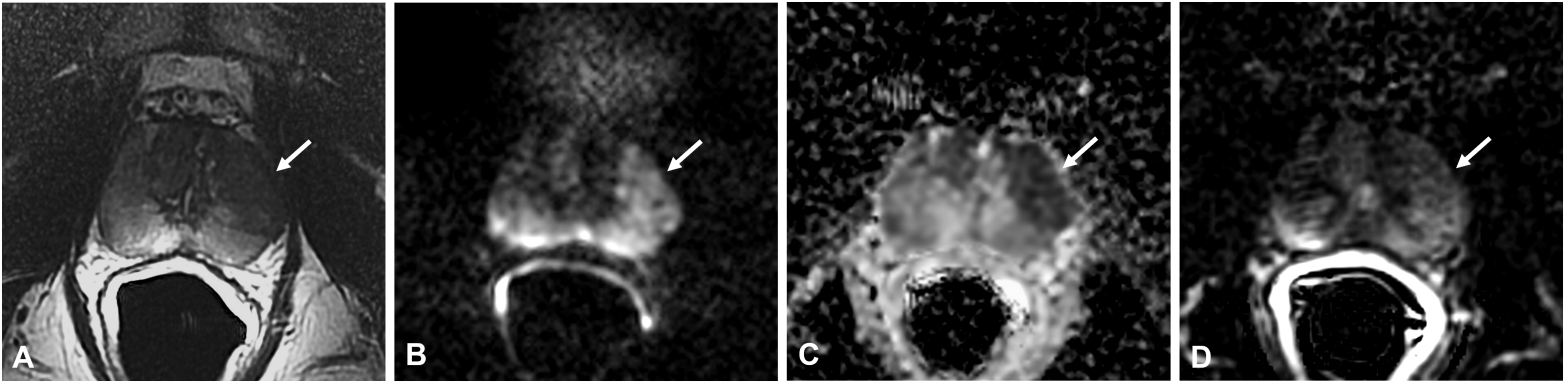
Non-specific granulomatous prostatitis in a 47-year-old patient with a PSA value of 9.81 ng/ml. The axial T2-weighted sequence shows a large hypointense area in the anterior left peripheral zone (PZ) with bulging of the glandular capsule (arrow in **A**) with diffuse hyperintensity on the DWI image (arrow in **B**), marked hypointensity on the ADC map (arrow in **C**), and diffuse mild enhancement on the DCE image (arrow in **D**).

**Figure 4:**
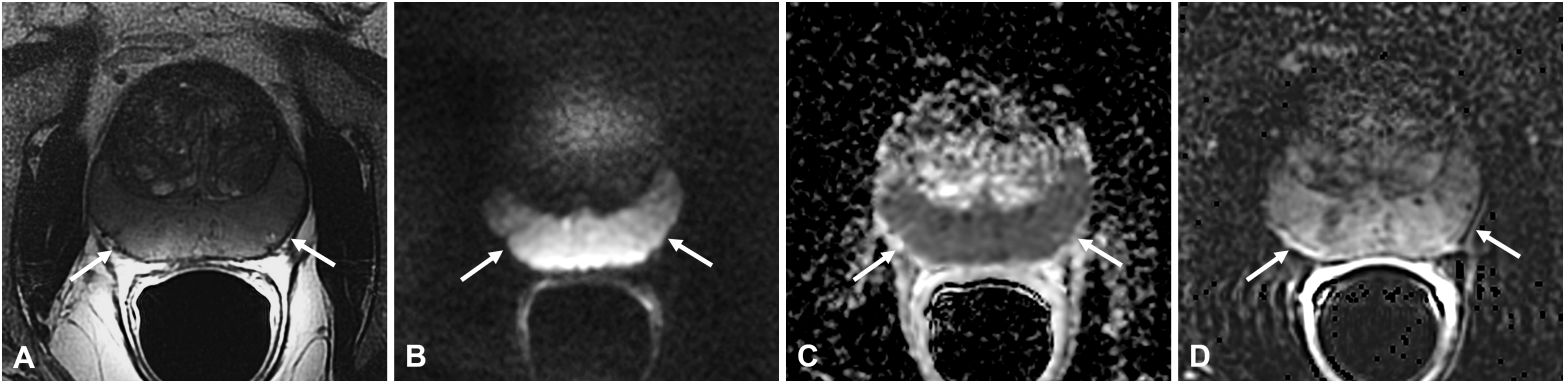
Non-specific granulomatous prostatitis in a 66-year-old patient with a PSA value of 15.92 ng/ml. The axial T2-weighted sequence shows diffuse hypointensity and thickening of the peripheral zone (PZ) with preservation of the glandular capsule and pseudocapsule (arrows in **A**). The entire PZ appears diffusely hyperintense on the DWI image (arrows in **B**) and markedly hypointense on the ADC map (arrows in **C**) with diffuse high contrast enhancement on the DCE image (arrow in **D**).

According to the literature, the typical pattern at the mpMRI shows a tumor-like appearance with hypointensity on T2w, hyperintensity on DWI, and low ADC values (6, 11–13) (**Table 4**; **Figures 2**–**4**).

In particular, on T2w, all lesions have lower signal intensity (SI) when compared to femoral head bone and demonstrate higher SI when compared with the obturator muscle (11).

On DWI, all lesions have higher signal intensity than the residual normal PZ (8). Many authors have demonstrated that the values of ADC in GP are lower than in high-grade PCa (5, 11, 13); thus, some authors have proposed using a nomogram that utilizes ADC values threshold to distinguish NSGP from PCa (14).

On DCE, almost all the lesions showed a moderate hyperenhancement after the administration of gadolinium-based contrast agent (5, 11, 13). However, in some patients with NSGP, DCE showed high and early enhancement followed by early wash-out (5), which is the typical pattern of PCa (15), while others highlighted a mild or scarce enhancement (5) (**Table 4**).

Some authors proposed that moderate hyperenhancement together with low ADC value may suggest the diagnosis of GP rather than PCa (13).

Some cases of diffuse GP may present with hyperintensity on T1w (16).

Morphologically, these lesions are nodular and circumscribed, with a diameter < 1.5 cm (12) (**Figure 2**) or between 1 and 3.5 cm (5) (**Figure 3**), usually confined to the PZ; more rarely can NSGP affect the whole PZ (**Figure 4**) or both the PZ and TZ; the involvement of only the TZ is an exception (5, 12) (**Table 4**).

Although it is infrequent, some cases in the literature report the presence of bulging and/or irregularity of the glandular capsule in NSGP while extra-capsular extension is usually absent (11, 12).

The xanthogranulomatous prostatitis (XGP) is a very rare subtype of NSGP and very few cases are reported in the literature. The distinctive histological feature is the presence of lipid-laden macrophages called “foamy histiocyte” in the inflammatory cell infiltrate.

The mpMRI showed isointensity on T1w, hypointensity on T2w, marked hyperintensity on DWI, low signal on ADC map, and hyperenhancement on DCE. Unlike NSGP, XGP has a patchy or diffuse involvement of the PZ with disappearance of the normal demarcation between PZ and TZ while the prostate capsule is always preserved (10) (**Table 4**).

#### 2.1.2 Infective granulomatous prostatitis and iatrogenic (post-surgical) granulomatous prostatitis

The cases reported in literature of infective GP are caused by some infectious agents such as virus-like herpes zoster, fungi like *Cryptococcus*, and bacteria like *Mycobacterium tuberculosis* and *Treponema pallidum* (4, 16) (**Table 1**).

The histological features are usually characterized by granulomas with epithelioid and multinucleated giant cell infiltration with caseous necrosis (4) (**Table 2**).

Among the iatrogenic causes are as follows: transurethral resection of the prostate (TURP) or the bladder (TURB), prostate biopsy, and open adenomectomy (6) (**Table 1**).

The most common etiology of specific GP is caused by a later complication of intravesical instillation of BCG; the BCG immunotherapy, given after TURB, is the most effective adjuvant treatment for intermediate and high-grade NMIBC (9).

Some studies proved that 75%–100% of the patients who undergo BCG instillations develop specific GP (17).

The appearance of tubercular GP is variable (16); in fact, three patterns can be identified: diffuse, nodular, and cystic (8).

The most common type is the diffuse pattern in which there is a heterogeneous, non-circumscribed, diffused involvement of the PZ with frequent extension in the TZ and the presence of capsular bulging, without invasion of peri-prostatic tissue (8, 12).

On mpMRI, diffuse pattern of non-necrotic GP shows low T2 signal intensity, similar to SI of bone marrow but lower than the SI of the normal PZ (8) (**Table 4**).

These lesions, typical of the acute phase, are associated with diffuse restriction on DWI, moderate or marked enhancement on DCE (4), and a decreased signal on the ADC map images (12); these features make them difficult to distinguish from cancer (**Table 4**).

The nodular pattern is characterized by the presence of solid nodules, polygonal in shape (8) and measuring > 1.5 cm (12), with marked hypointensity of signal in T2w sequences and isointensity of signal on T1w sequences if compared with obturator muscle (8) (**Table 4**).

All nodular lesions show higher signal intensity on DWI, because DWI reflects the cell density increased by the presence of lymphocytes during acute inflammation (8), and lower SI on ADC when compared to the normal PZ (9) (**Table 4**).

If there are no necrotic areas, the contrast enhancement is inhomogeneous, early and prolonged (5, 9).

**Figure 5:**
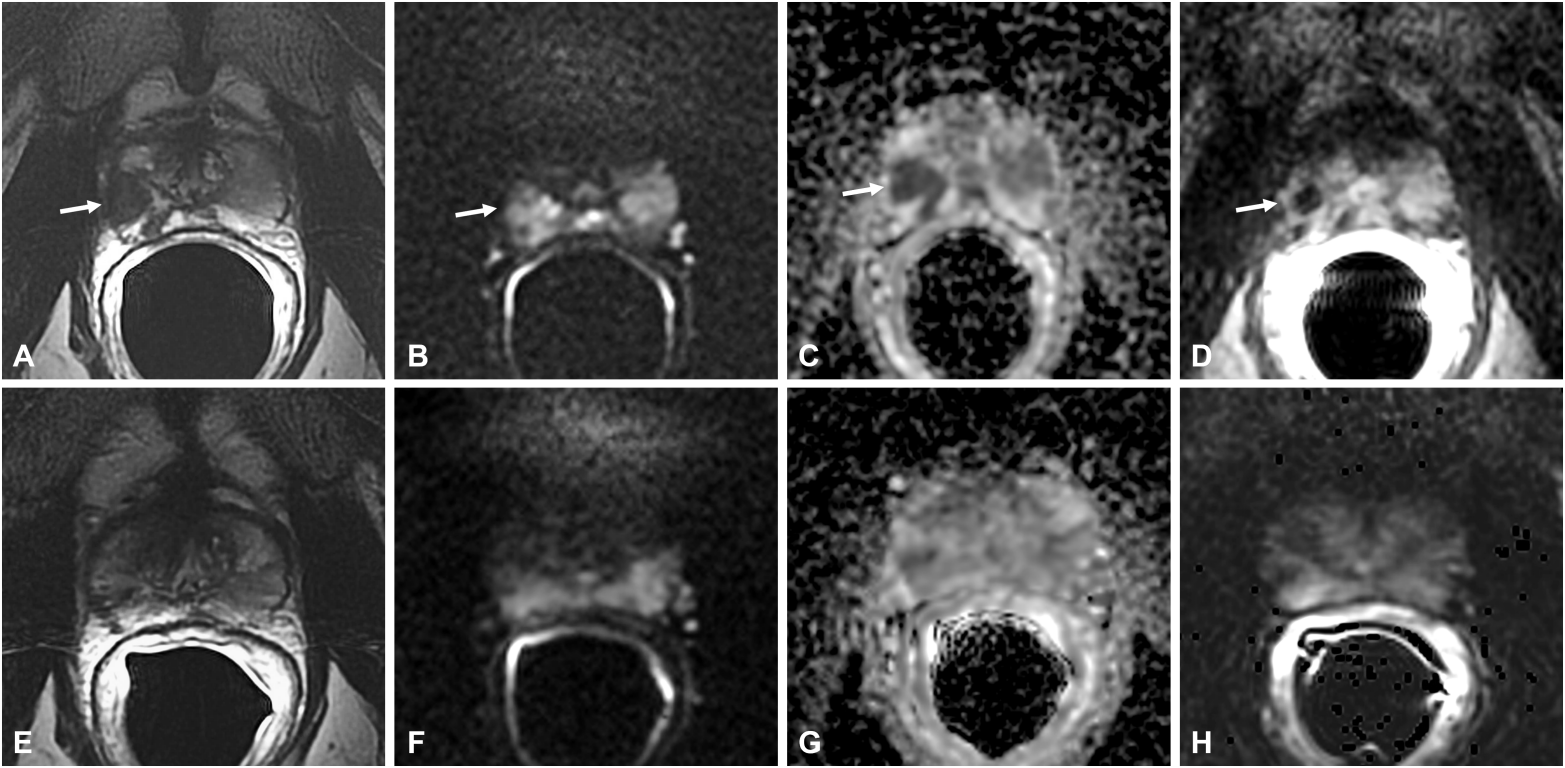
Bacillus Calmette-Guérin-induced granulomatous prostatitis in a 58-year-old patient with a PSA value of 1.17 ng/ml. The first mpMRI at diagnosis showed diffuse alteration of the peripheral zone (PZ) with a hypointense nodule in the right middle lobe in the T2-weighted sequence (arrow in **A**) with hyperintensity on the DWI image (arrow in **B**), marked hypointensity on the ADC map (arrow in **C**), and typical “ring enhancement” on the DCE image (arrow in **D**). The corresponding sequences of the 5-month mpMRI follow-up **(E–H)** showed almost complete resolution of the aforementioned findings.

**Figure 6:**
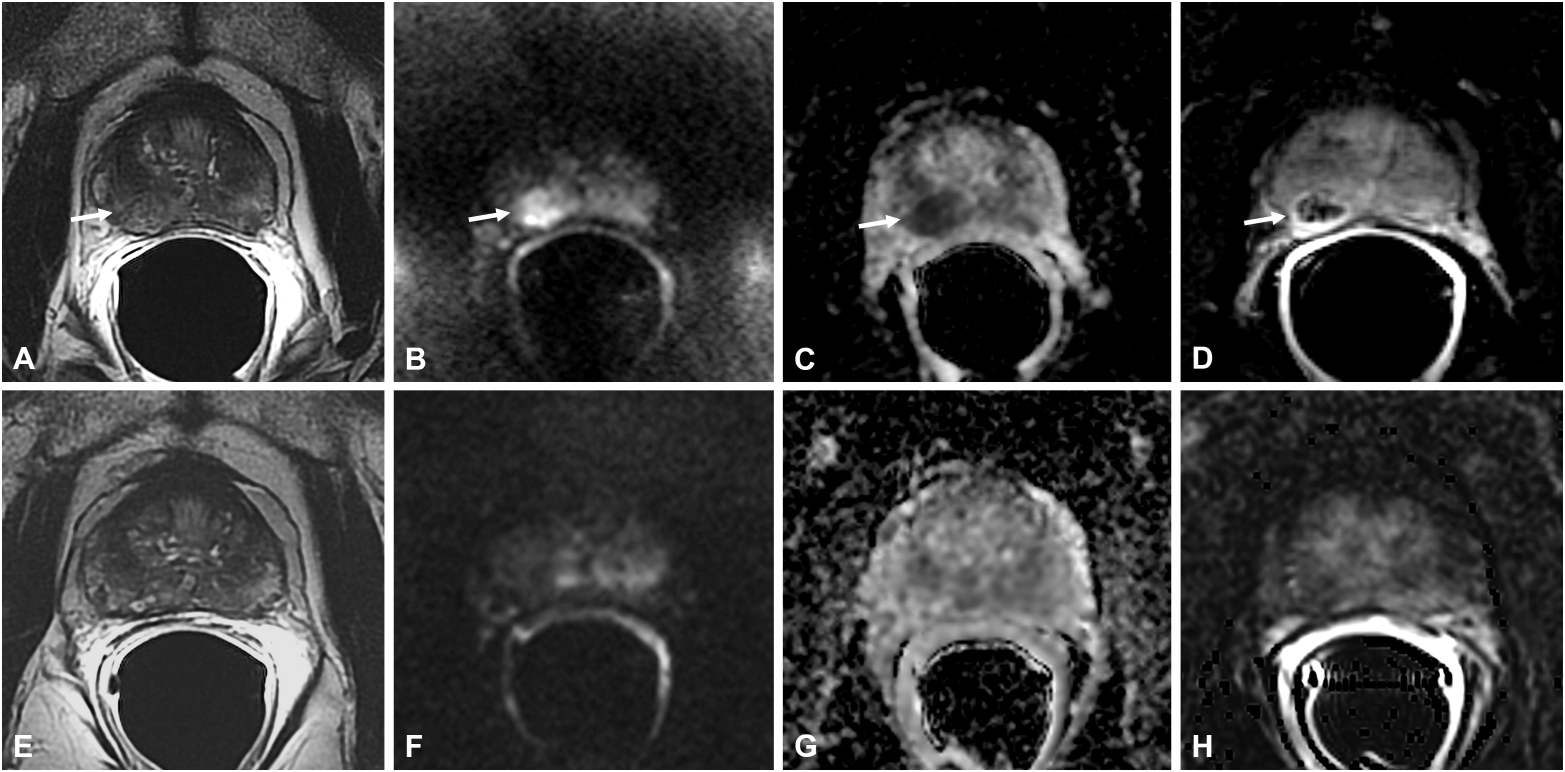
Bacillus Calmette-Guérin-induced granulomatous prostatitis in a 53-year-old patient with a PSA value of 5 ng/ml. The first mpMRI at diagnosis showed diffuse alteration of the peripheral zone (PZ) with an inhomogeneous hypointense nodule in the right middle lobe in the T2-weighted sequence (arrow in **A**) with hyperintensity on the DWI image (arrow in **B**), marked hypointensity on the ADC map (arrow in **C**), and typical “ring enhancement” on the DCE image (arrow in **D**). The corresponding sequences of the 7-month mpMRI follow-up **(E–H)** showed complete resolution of the aforementioned findings.

The cystic pattern with mural nodules is caused by central caseous necrosis that manifests with the tubercular granuloma (16). Therefore, in necrotic GP, central necrosis (caseation) is hyperintense on T2w sequences, with marked signal restriction on high b-value DWI, low ADC value, and total lack of contrast enhancement (4, 6), while mural nodules show hypointensity on T2w sequences with contrast enhancement on DCE imaging (8) (**Table 4**; **Figures 5**, **6**).

Thus, on DCE sequences, these lesions have a typical enhancement behavior characterized by an early and prolonged peripheral rim enhancement with avascular core due to caseous necrosis, already described as “ring enhancement” (5, 15, 16) (**Figures 5**, **6**).

Recently, Lee et al. (18) focused on the multiphase contrast enhancement pattern of BCG-induced GP lesions in a cohort of 24 patients. They found three typical patterns of vascularity based on sequential changes and histological findings: pattern A demonstrated diffuse enhancement, pattern B demonstrated lesions with ring enhancement, and pattern C demonstrated poor rim enhancement. Types A and B are regarded as acute stages, which show diffusion restriction on DWI and intense enhancement, and the difference is, respectively, the absence and presence of a well-defined poorly enhancing area in types A and B; type C is a chronic lesion showing poor enhancement and a low signal intensity on high b-value DWI.

#### 2.1.3 Associated with systemic granulomatous disease

Some articles in the literature report a suspected correlation between GP and systemic disease such as psoriasis, sarcoidosis, rheumatoid arthritis, Wegener’s granulomatosis, polyarteritis nodosa, and Churg–Strauss syndrome (**Table 1**). The histopathological features depend on the primary granulomatous disease (**Table 2**).

Usually, it is a nodular lesion that can involve both the peripheral and transition zones with a capsular irregularity. mpMRI shows a low signal intensity on T2w, a significant signal restriction on DWI with low ADC and a diffuse contrast enhancement on DCE (**Table 4**).

It is interesting to note that there could be a link between GP and systemic granulomatous diseases, because these conditions are characterized by an abnormal response of the immune system that could play a direct role in their pathogenesis (19).

## 3 Discussion and conclusion

GP is a relatively rare chronic inflammatory disease of the prostate (5), which represents approximately 3.3% of all benign conditions of the gland (6), whose incidence is increasing due to the growth of endourological surgical interventions and the adoption of intravesical instillation of BCG in patients with NMIBC (6).

Despite being a benign condition, GP represents one of the main adenocarcinoma-mimicking lesions and in most cases produces PI-RADS ≥ 3 findings at mpMRI, placing a high suspicion of PCa. In a cohort of 105 biopsied PI-RADS 5 lesions, Pepe et al. found six (5.7%) GP, with five non-specific GP and one specific GP secondary to prostatic *M. tuberculosis* (20).

In this article, we have tried to identify the peculiar characteristics of the GP at mpMRI, through a narrative review of the literature, with the aim of improving the differential diagnosis with adenocarcinoma and avoid the use of prostate biopsy as much as possible.

NSGP, including XGP, is a very rare inflammatory condition and radiological reports at mpMRI are too few. According to the literature, we have seen that the typical pattern of NSGP at the mpMRI shows nodular lesions, confined to the PZ, with a tumor-like appearance (6, 11–13). Although DCE images are not specific, moderate or scarce hyperenhancement together with a very low ADC value may suggest the diagnosis of GP rather than PCa (13); therefore, in these cases, and in agreement with the referring urologist, a wait-and-see attitude could be proposed with a re-evaluation on mpMRI after a few months to value the possible self-resolution of the inflammatory picture (19). However, in cases where DCE images showed high and early enhancement followed by early wash-out (5), there is the presence of bulging and/or irregularity of the glandular capsule, or there are no clear parameters to differentiate NSGP from PCa, the use of biopsy is mandatory.

In most cases, NSGP resolves spontaneously without treatments with normalization of PSA level (19); however, careful follow-up is required after this diagnosis in order to exclude the coexistence of an occult PCa.

Nowadays, intravesical BCG instillation is widely used as a treatment for non-muscle invasive bladder cancer after transurethral resection though the immune system activation and the induction of inflammatory response (21). The intraprostatic reflux of contaminated urines from urethra can cause the BCG-induced GP and it usually involves the PZ because of the obtuse angle between the PZ and the urethra (18).

The BCG-induced GP can present variable appearances, probably based on the different stages of the disease. At a certain stage of the development of the disease, some cavitated nodules with a characteristic cystic pattern appear. This pattern consists of hyperintensity on T2 sequences, with marked hyperintensity on high b-value DWI and low ADC value; on DCE sequences, there is a typical enhancement behavior characterized by an early and prolonged peripheral rim enhancement with avascular core. Kawada et al. (15) first analyzed the multiphase contrast enhancement pattern of BCG-induced GP lesions on gadolinium-enhanced MR images showing this characteristic appearance defined “ring enhancement”. The histological analysis confirmed the correspondence between the granulomatous tissue with central caseation necrosis and the ring enhancement area. The authors concluded that this appearance could be considered characteristic of the BCG-induced GP and could be useful to differentiate it from PCa. More recently, Lee et al. (18) attempted to differentiate PCa from BCG-induced GP on the basis of the multiphase contrast enhancement pattern. During the acute phase of the disease, the diffuse enhancement pattern, also called pattern A, prevails and the diagnosis of PCa is more challenging; thus, follow-up or a biopsy is needed. A possible differentiation with PCa can be made in the presence of lesions with ring enhancement, in pattern B, or chronic lesions, in pattern C.

Recognizing the key role of the DCE sequence is fundamental for obtaining the proper identification of caseating granulomas as their high values in DWI sequence could lead to an incorrect diagnosis; this observation highlights the limits of the biparametric MRI protocol in the evaluation of this pathology and suggests the need for the multiparametric MRI protocol in patients with recent intravesical BCG instillation.

Furthermore, a mpMRI follow-up of prostatic lesions, as an alternative to a biopsy, is suggested in patients with suspicious lesions and a history of vesical instillation of BCG for bladder carcinoma as these granulomas can decrease in size, thus suggesting an antitubercular therapy, when required (9). As in the study of Lee et al. (18), the date interval for follow-up after the first diagnosis varied considerably in our study, thus denying a correlation between duration and stage of the disease. No further and precise indications can be extracted from the literature regarding this.

Finally, the present study outlined the limits of the correct assignment of the PI-RADS score in evaluating the radiological findings of benign inflammatory conditions, the proper classification of which can be obtained from its complete clinical context. Therefore, the need to integrate the PI-RADS system in the evaluation of specific benign conditions such as inflammatory diseases can be argued.

The main limitation of the present study is the lack of large series studies on this topic, owing to the rarity of the disease. In fact, mainly case reports and small case series are reported in the literature; thus, a detailed statistical analysis has not been possible.

As a result of the review of the literature, we can conclude that mpMRI of the prostate may play a key role in differentiating BCG-induced GP from adenocarcinoma of the prostate on the basis of the correct evaluation of the typical “ring enhancement” of the prostate lesions on the multiphase contrast-enhanced MRI, in the presence of specific clinical context; an mpMRI follow-up of prostatic lesions can be safely carried out. Conversely, the correct diagnosis of other cases of non-necrotic GP (including NSGP, XGP, and diffuse or nodular BCG-induced GP) is not possible on the basis of the mpMRI features, even when considering the PSA values; a targeted biopsy remains the mandatory approach.

## Author contributions

CG conceived, guided, and revised the manuscript. BR reviewed the literature and wrote the original draft of the project. CF, BC, and FC contributed to the literature review and description of MRI features. LB, RS, MD, and FG provided experience in clinical and surgical parts. EB and MF helped in histopathological review. All authors contributed to the article and approved the submitted version.
